# Perinatal outcomes associated with magnesium sulfate for preeclampsia without severe features

**DOI:** 10.1002/pmf2.70309

**Published:** 2026-05-23

**Authors:** Samantha E. Howell, Christina T. Blanchard, Morgan Jenkins, Grace Lee, Macie L. Champion, Michelle Lu, Ashley N. Battarbee, Brian M. Casey, Akila Subramaniam

**Affiliations:** ^1^ Department of Obstetrics and Gynecology Center for Women's Reproductive Health at the University of Alabama at Birmingham Birmingham Alabama USA; ^2^ Department of Obstetrics and Gynecology Division of Maternal Fetal Medicine University of Wisconsin‐Madison Madison Wisconsin USA; ^3^ University of Alabama at Birmingham Heersink School of Medicine Birmingham Alabama USA; ^4^ Department of Obstetrics and Gynecology Division of Gynecologic Oncology The University of Chicago Chicago Illinois USA; ^5^ Department of Obstetrics and Gynecology Division of Maternal Fetal Medicine Center for Women's Reproductive Health at the University of Alabama at Birmingham Birmingham Alabama USA; ^6^ Maternal Fetal Medicine, Novant Health Charlotte North Carolina USA; ^7^ Moontower Midwifery and Wellness Austin Texas USA; ^8^ Department of Obstetrics Gynecology and Reproductive Sciences West Virginia University Medicine Golisano Children's Morgantown West Virginia USA

**Keywords:** magnesium sulfate, neonatal acidemia, neonatal outcomes, NICU admission, preeclampsia, seizure prophylaxis

## Abstract

**Objective:**

To evaluate neonatal outcomes in patients with preeclampsia without severe features who received magnesium for seizure prophylaxis.

**Methods:**

Retrospective cohort study of patients with preeclampsia without severe features delivering ≥32 weeks’ gestation at our institution (November 2021–May 2023). The primary outcome was a composite of adverse neonatal outcomes (neonatal intensive care unit [NICU] admission, umbilical artery pH < 7.1, umbilical artery base deficit ≥12, 5‐min Apgar <3, neonatal death, culture proven sepsis, and mechanical ventilation). Secondary outcomes included the individual components of the neonatal composite and a maternal bleeding composite. Patients were categorized into those who received intrapartum magnesium and those who did not. Baseline characteristics and outcomes were compared between groups. Multivariable logistic regression estimated the association between magnesium and outcomes, adjusting for characteristics that differed between groups.

**Results:**

Of 5327 individuals included in our analysis, 583 met study criteria—439 (75%) received magnesium and 144 (25%) did not. Patients who received magnesium were more likely to have public insurance, medical comorbidities, antenatal aspirin use, earlier gestational age (GA) at delivery, and chorioamnionitis. The odds of composite adverse neonatal outcome were higher in patients who received magnesium (53% vs. 29%; adjusted odds ratio [aOR], 1.85; 95% confidence interval [CI], 1.13–3.04) than those who did not. This was primarily driven by increased odds of NICU admission (aOR, 2.46; 95% CI, 1.46–4.14). When excluding NICU admission from the neonatal composite, there was a significant reduction in the neonatal adverse composite among the magnesium cohort (aOR, 0.43; 95% CI, 0.21–0.88). In addition, the odds of neonatal acidemia were also lower in those who received magnesium (aOR, 0.36; 95% CI, 0.15–0.88).

**Conclusion:**

While we found an increase in overall adverse neonatal outcomes in patients receiving magnesium for seizure prophylaxis for preeclampsia without severe features, we also noted discordant findings of decreased neonatal acidemia and neonatal morbidity when excluding NICU admission. As there is no consensus for intrapartum magnesium administration for patients with preeclampsia without severe features, these results suggest further study of risks and benefits of intrapartum magnesium sulfate is warranted.

## 1 INTRODUCTION

Preeclampsia occurs in 2%–8% of pregnancies globally and represents a major cause of maternal morbidity, with 16% of maternal deaths worldwide attributed to hypertensive disorders of pregnancy [1, 2, 3]. Eclampsia is a rare event but contributes significantly to maternal death in patients with hypertensive disorders of pregnancy, particularly in low‐resource settings [4]. Administration of magnesium sulfate intrapartum and for 24 hours postpartum decreases eclamptic seizures by approximately 58% [5] and is currently recommend by the American College of Obstetricians and Gynecologists (ACOG) for patients with preeclampsia with severe features for seizure prophylaxis [4].

The benefits of intrapartum magnesium administration for patients with preeclampsia without severe features are less clear. ACOG currently recommends that the administration of magnesium for individuals with preeclampsia without severe features be individualized and determined by the physician or institution considering the risks and benefits of magnesium [4]. This has led to practice variation among clinicians, both in the United States and worldwide. In fact, a multicountry study by Long et al. suggests that magnesium is still used globally for preeclampsia without severe features [6].

Despite the significant reduction in seizures with magnesium sulfate administration, there are multiple potential adverse effects of magnesium administration. Side effects of magnesium include flushing, nausea, vomiting, headache, muscle weakness, dizziness, and drowsiness are thought to occur in approximately 25% of patients who receive magnesium [5]. Albeit rare, magnesium has the potential for toxicity, which can lead to loss of deep tendon reflexes, respiratory paralysis, alteration of cardiac conduction, and ultimately cardiac arrest [7]. While data are conflicting, some studies report an increased risk of postpartum hemorrhage with magnesium administration [8, 9, 10]. Considering the potential for significant risks, side effects, and adverse events associated with magnesium, appropriate candidates for administration of magnesium should be carefully selected. Given the need for more data to guide this decision to administer magnesium prophylaxis, we aimed to evaluate neonatal outcomes, as well as maternal outcomes, of patients who received magnesium for seizure prophylaxis in the setting of preeclampsia without severe features.

## 2 METHODS

After receiving approval by the University of Alabama at Birmingham (UAB) international review board (IRB #‐300002492), we performed a retrospective cohort study of patients who delivered at our institution between November 1, 2021 and May 31, 2023. The STROBE (Strengthening the Reporting of Observational Studies in Epidemiology) guidelines for cohort studies were followed.

Patients who delivered during the aforementioned timeframe and were diagnosed with preeclampsia without severe features > 32 weeks gestational age (GA) were included. Preeclampsia was diagnosed as defined by the ACOG and diagnosis codes were used for inclusion and exclusion [4]. Both singleton and twin gestations were included. Antenatally diagnosed fetal anomalies and patients delivering <32 weeks gestation were excluded. We specifically chose this gestational age of 32 weeks given that magnesium is recommended for fetal neuroprotection at less than 32 weeks and also used for this alternate indication in this population. Our institution does not use magnesium as a tocolytic and thus, if intrapartum magnesium was administered beyond 32 weeks’ gestation, it was for seizure prophylaxis alone.

At our institution, patients with preeclampsia at a gestational age beyond 32 weeks receive a 6 g bolus over 20 min followed by a constant infusion at a rate of 2 g/h during labor. The rate of magnesium infusion is adjusted for those with renal disease. If the creatinine is between 1.1 and 1.5, the 6 g bolus is followed by a constant infusion at a rate of 1 g/h with magnesium and creatinine serum levels obtained every 4–6 h and close monitoring of urine output. If the creatinine is >1.5, a 4 g bolus of magnesium is given but continuous infusion is not administered. Magnesium and creatinine serum levels are trended, and magnesium is re‐bolused as needed to reach a therapeutic level (4.8–8.4). Magnesium is continued in all patients for 24 h following delivery. Of note, at our institution, the receipt of intrapartum magnesium for seizure prophylaxis for preeclampsia without severe features is per clinician discretion. Historically at our institution, intrapartum magnesium sulfate was given to all patients with a diagnosis of preeclampsia without severe features. Restructuring of the labor and delivery service line in 2021 led to more patients being managed by the Division of Women's Reproductive Healthcare as opposed to the Division of Maternal Fetal Medicine (MFM). The MFM division generally chooses to use magnesium for preeclampsia without severe features based on the small benefit noted in the Magpie trial, while the generalist division defers to the individual clinician's discretion. As such, after the change of service line in 2021 there became more variation in the use of intrapartum magnesium sulfate for patients with preeclampsia without severe features >32 weeks gestation.

Our primary outcome was an adverse neonatal composite which included neonatal intensive care unit (NICU) admission, arterial blood gas pH < 7.1, base deficit ≥12 (defined as the absolute value of a negative base excess with increasing positive values reflecting worsening metabolic acidosis), 5‐min Apgar < 3, neonatal death, culture proven sepsis, and need for mechanical ventilation. Of note, at our institution, receipt of magnesium sulfate is not an indication for admission to NICU. Secondary outcomes were individual components of the neonatal composite, birthweight, NICU length of stay, 1‐min Apgar, 5‐min Apgar, a maternal bleeding composite (including estimated blood loss [EBL] > 1 L and transfusion of packed red blood cells [RBCs]), individual components of the maternal bleeding composite, and the difference in hemoglobin from predelivery to postdelivery. The majority of these maternal outcomes were chosen given the conflicting data about the association of magnesium and postpartum hemorrhage. For multiple gestations, a pregnancy was classified as having the outcome of interest if at least one neonate met the specified criteria. The birthweight of fetus A was reported.

Our institution's electronic medical record (Cerner IMPACT) contains all information for patient encounters anywhere within the hospital system. Baseline characteristics, primary, and secondary outcomes were obtained from a combination of individual chart review and validated coded data extraction as detailed in Lu et al. and Champion et al. [11, 12]. Chart review was performed by research team members (maternal‐fetal medicine faculty and fellows, obstetrics and gynecology residents, and third‐year medical students) trained by the primary and senior authors (S.E.H., M.L.C., and A.K.S.) using prespecified rules for defining each variable. Furthermore, individual chart review was used to confirm that patients with preeclampsia with severe features were not included. The individual variables that were obtained via chart review and code extraction are found in Appendix 1 of Lu et al. and available online at http://links.lww.com/AOG/C407. [11].

Patients with a diagnosis of preeclampsia without severe features were categorized into two groups: those who received intrapartum magnesium for seizure prophylaxis and those who did not. Differences in baseline characteristics, primary, and secondary outcomes were compared between groups using the chi‐square test of association or Fisher's exact test for categorical variables and Student's *t*‐tests for continuous variable as appropriate. Multivariable logistic regression was used to estimate odds ratios (ORs) and adjusted odds ratios (aORs) with 95% confidence intervals (CIs) for primary and secondary outcomes, using patients who did not receive magnesium as the referent group. Significantly different baseline characteristics (defined as *p* < 0.05) were considered as covariates to calculate aOR; in the case of non‐convergence due to low incidence of a significant covariate, this covariate was excluded to prevent over‐fitting. Sensitivity analyses were performed to assess the impact of individual components on the composite neonatal outcome. These included a composite excluding NICU admission and a composite excluding both NICU admission and neonatal sepsis.

Missingness was assessed for all variables. While most variables had minimal missing data (<5%), pH < 7.1, base deficit ≥12, and birthweight had higher levels of missingness (12.3%, 12.7%, and 11.7%). Analyses were conducted using available data. Since this study is hypothesis generating, imputation for missing data was not performed. All statistical analyses were performed by the University of Alabama at Birmingham's Center for Women's Reproductive Health Biostatistics and Data Management Core using SAS, version 9.4 (SAS Institute Inc). The level of statistical significance was set at 0.05. A priori power calculation was not performed given the study period start and end date was a sample size of convenience.

## 3 RESULTS

**FIGURE 1:**
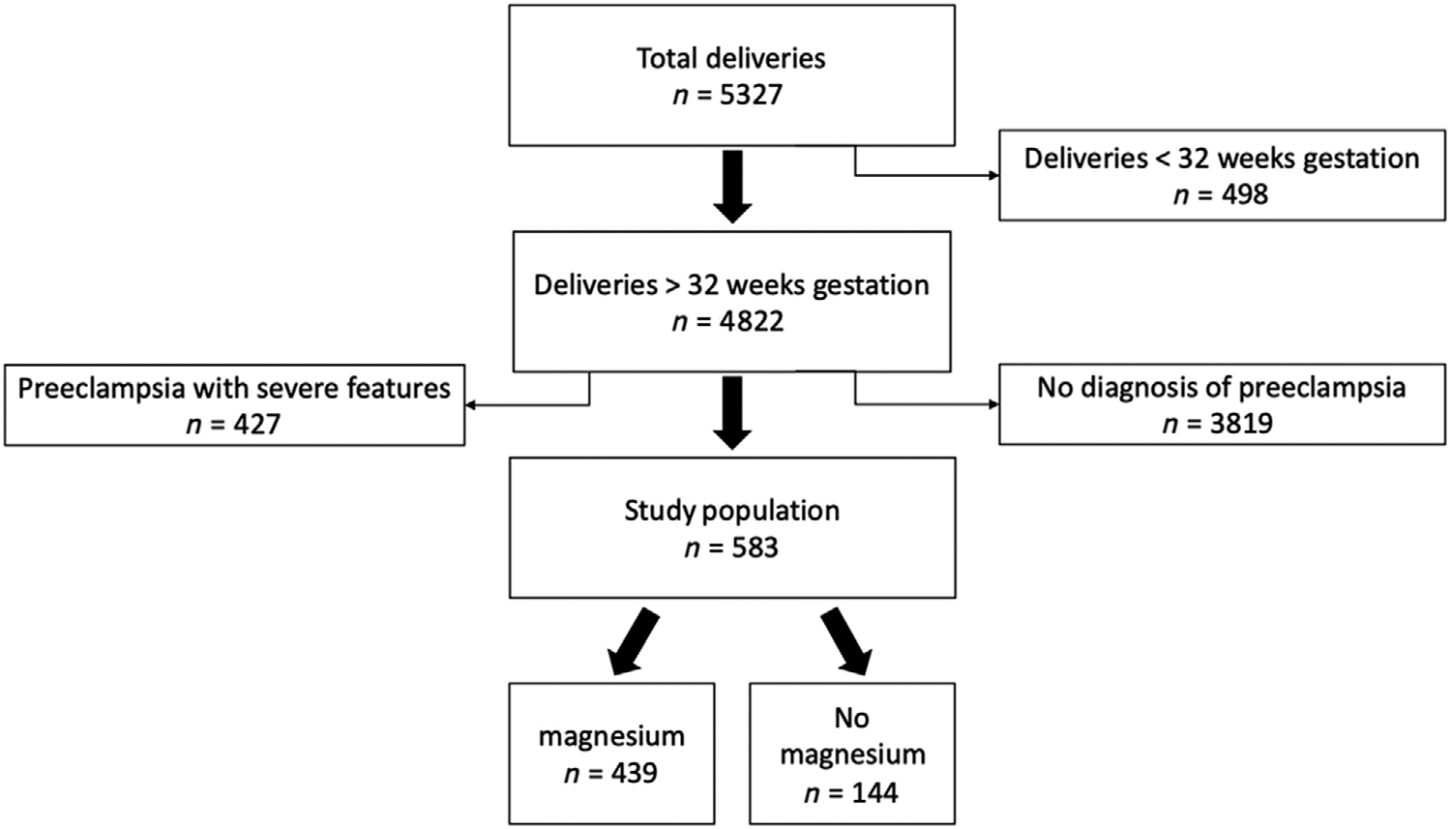
Exclusion criteria: this figure depicts the inclusion and exclusion criteria used to derive the final study population.

Of 5327 deliveries that occurred at our institution between November 1, 2021 and May 31, 2023, 498 were excluded due to delivery prior to 32 weeks gestational age. There were 3819 patients in the study period who were not diagnosed with preeclampsia and excluded; 427 patients with preeclampsia with severe features were excluded. Ultimately, 583 patients were included in our study (Figure 1). Of these patients, 439 (75%) received intrapartum magnesium and 144 patients did not receive intrapartum magnesium (25%).

Baseline characteristics are presented in Table 1. Groups were different in multiple characteristics including insurance status, marital status, prepregnancy body mass index (BMI), alcohol use during pregnancy, aspirin use in pregnancy, gestational age at delivery, diagnosis of chorioamnionitis, the presence of other comorbidities including chronic hypertension (CHTN), and pregestational diabetes (Table 1). Specifically, patients who received magnesium had a higher incidence of government‐funded insurance, a higher BMI at intake exam (34.7 vs. 30.8), alcohol use, aspirin use, chronic hypertension, and gestational or pregestational diabetes. Patients who received magnesium additionally delivered at an earlier gestational age and were more frequently diagnosed with chorioamnionitis (Table 1). Missingness for baseline covariates included in Table 1 was low (<5% for all variables).

**TABLE 1:** Baseline characteristics.

Characteristic	Magnesium (*n* = 439)	No magnesium (*n* = 144)	*p* value
Maternal age at delivery (years)	29.1 ± 6.5	29.0 ± 6.1	0.89
Self‐reported race/ethnicity^a^			0.21
Black	202 (46.0)	57 (39.6)	
White	142 (32.4)	58 (40.3)	
Hispanic/other	95 (21.6)	29 (20.1)	
Government‐funded insurance	**283 (64.8)**	**70 (48.6)**	**<0.001**
Married	**156 (35.9)**	**65 (45.1)**	**0.049**
Prepregnancy BMI (kg/m^2^)	**34.7 ± 10.5**	**30.8 ± 8.8**	**<0.001**
Singleton pregnancy	418 (95.2)	138 (95.8)	0.76
Tobacco use in pregnancy	49 (11.5)	13 (9.1)	0.42
Alcohol use in pregnancy	**37 (8.8)**	**24 (17.3)**	**0.006**
Drug use in pregnancy	12 (2.9)	3 (2.1)	0.77
Aspirin use during pregnancy	**201 (45.8)**	**46 (31.9)**	**0.004**
Chronic hypertension	**173 (39.4)**	**42 (29.2)**	**0.027**
Pregestational diabetes mellitus	**66 (15.0)**	**9 (6.3)**	**0.006**
Maternal cardiac disease	34 (7.7)	8 (5.6)	0.38
Chronic kidney disease	15 (3.4)	1 (0.7)	0.14
Gestational diabetes	71 (16.2)	14 (9.7)	0.06
Gestational age at delivery (weeks)	**36.9 ± 2.0**	**38.2 ± 1.7**	**<0.001**
Predelivery hemoglobin (g/dL)	11.2 ± 1.4	11.2 ± 1.4	0.97
Cesarean delivery	179 (40.9)	57 (39.9)	0.83
Chorioamnionitis	**10 (2.3)**	**10 (6.9)**	**0.014**
Delivery anesthesia			0.45
General	8 (1.8)	4 (2.8)	
Neuraxial	279 (63.6)	81 (56.3)	
IV narcotics or no anesthesia	106 (24.2)	42 (29.2)	
Other or unclear	46 (10.5)	17 (11.8)	
Bilateral tubal ligation performed	45 (10.3)	8 (5.6)	0.09
Delivery year			0.81
2021	32 (7.3)	9 (6.3)	
2022	307 (69.9)	99 (68.8)	
2023	100 (22.8)	36 (25.0)	

*Note*: The variables/outcomes that are statistically significant are bolded.

^a^ In our institutions’ medical record, patients self‐report race. The available categories include White, Black, Hispanic, and Other (Asian, Islander, etc.).

^b^ Missing data <5% for all variables.

Primary and secondary outcomes are presented in Table 2. We observed that patients who received magnesium had a nearly twofold increased odds of the neonatal composite outcome compared to those who did not (53% vs. 29%; OR, 2.87, 95% CI, 1.91–4.31). This relationship persisted in multivariate analysis even after adjusting for gestational age at delivery, public insurance, marital status, BMI, alcohol use, aspirin use, chronic hypertension, pregestational diabetes, and chorioamnionitis (aOR, 1.85; 95% CI, 1.13–3.04) (Table 2). The increase in the neonatal adverse composite observed in patients who received magnesium was largely driven by an increase in NICU admission. Even with adjusting for gestational age among other covariates, patients who received magnesium had a more than twofold increased odds of NICU admission compared to those who did not, which was significant in univariate and multivariate analyses (aOR, 2.46; 95% CI, 1.46–4.14, Table 2). Of note, the length of NICU stay was similar between groups (11.1 days magnesium vs. 12.6 days no magnesium, *p* = 0.60). Despite increased overall neonatal composite outcomes and NICU admissions, however, there was a significantly decreased risk of pH < 7.1 in those who received magnesium (4% vs. 9%; aOR, 0.36; 95% CI, 0.15–0.88). There were no other significant differences in components of the neonatal composite including base deficit ≥12, 5‐min Apgar <3, neonatal death, culture proven sepsis, or mechanical ventilation between groups. When a sensitivity analysis was performed excluding NICU admission from the neonatal composite, there was a significantly reduced odds of the neonatal adverse composite among the magnesium cohort (7% vs. 11%; aOR, 0.43; 95% CI, 0.21–0.88). These results persisted in a sensitivity analysis excluding NICU admission and sepsis from the neonatal composite. Lastly, there was no difference in maternal outcomes between the two groups (Table 2).

**TABLE 2:** Comparison of primary and secondary outcomes by magnesium administration.

Outcome	Magnesium (*n* = 439)	No magnesium (*n* = 144)	OR (95% CI) or *p* value as applicable	aOR (95% CI)^a^ No magnesium as referent group
Neonatal composite	**234 (53.3)**	**41 (28.5)**	**2.87 (1.91–4.31)**	**1.85 (1.13–3.04)**
NICU admission	**225 (51.3)**	**33 (22.9)**	**3.54 (2.30–5.44)**	**2.46 (1.46–4.14)**
pH < 7.1^b^, ^e^	**16 (4.2)**	**12 (9.3)**	**0.43 (0.20–0.93)**	**0.36 (0.15–0.88)**
Base deficit > /12^b^, ^f^	4 (1.1)	3 (2.3)	0.44 (0.10–2.00)	0.60 (0.10–3.54)
5‐min Apgar < 3^c^	3 (0.7)	3 (2.1)	0.32 (0.06–1.60)	0.19 (0.03–1.15)
Neonatal death	0 (0.0)	0 (0.0)	–	–
Culture proven sepsis	3 (0.7)	0 (0.0)	*p* > 0.99	–
Mechanical ventilation^d^	8 (1.8)	1 (0.7)	2.65 (0.33–21.39)	1.30 (0.15–11.34)
Secondary outcomes				
1‐min Apgar	**6.7 ± 2.2**	**7.2 ± 2.0**	***p* = 0.020**	–
5‐min Apgar	**8.3 ± 1.2**	**8.6 ± 1.3**	***p* = 0.018**	–
Birthweight	**2814.5 ± 657.7**	**3160.4 ± 574.2**	***p* < 0.001**	–
NICU length of stay (days)	11.1 ± 9.7	12.6 ± 6.7	*p* = 0.60	
Maternal bleeding composite	51 (11.6)	20 (13.9)	0.82 (0.47–1.42)	0.88 (0.47–1.66)
EBL > 1 L^b^	15 (3.5)	8 (5.7)	0.60 (0.25–1.44)	0.63 (0.23–1.73)
Transfusion of PRBCs	39 (8.9)	19 (13.2)	0.64 (0.36–1.15)	0.71 (0.36–1.40)
Change in hemoglobin	1.9 ± 1.3	1.8 ± 1.3	*p* = 0.71	–
Sensitivity analyses				
Neonatal composite without NICU	30 (6.8)	16 (11.1)	0.59 (0.31–1.11)	**0.43 (0.21–0.88)**
Neonatal composite without NICU admission and sepsis	28 (6.4)	16 (11.1)	0.55 (0.29–1.04)	**0.40 (0.20–0.83)**

*Note*: The variables/outcomes that are statistically significant are bolded.

Abbreviations: aOR, adjusted odds ratio; BMI, body mass index; CI, confidence interval; CHTN, chronic hypertension; DM, diabetes mellitus; EBL, estimated blood loss; GA, gestational age; NICU, neonatal intensive care unit; OR, odds ratio; PRBC, packed red blood cells.

^a^ Adjusted for public insurance, married, BMI (as continuous variable), alcohol use, aspirin use, CHTN, pregestational DM, GA at delivery, and chorioamnionitis.

^b^ Adjusted for public insurance, married, BMI, alcohol use, aspirin use, CHTN, pregestational DM, and GA at delivery.

^c^ Adjusted for public insurance, married, BMI, aspirin use, CHTN, pregestational DM, GA at delivery, and chorioamnionitis.

^d^ Adjusted for public insurance, married, BMI, aspirin use, CHTN, pregestational DM, and GA at delivery.

^e^ Missing in 72 cases (12.3%).

^f^ Missing in 74 cases (12.7%).

^g^ Missing in 68 cases (11.7%).

## 4 DISCUSSION

We demonstrated an increased risk of adverse neonatal outcomes, primarily NICU admission, in patients with preeclampsia without severe features who received magnesium for seizure prophylaxis compared to those who did not. Surprisingly, however, rates of neonates with pH < 7.1 were significantly lower among this group. When excluding NICU admission from the neonatal composite, magnesium use was also associated with decreased odds of neonatal morbidity. These discordant findings persisted despite adjusting for covariates different between the groups at baseline. There was no difference in other secondary outcomes including neonatal death, Apgar scores, birthweight, or maternal bleeding composite. The discordant results of increased NICU admission but decreased rates of neonatal morbidity and acidemia with pH < 7.1 prompts the question of whether there is a protective effect to the neonate from magnesium. These findings warrant further investigation to delineate the true benefits and risks of magnesium sulfate for patients with preeclampsia without severe features. Of note, our findings are reassuring in terms of maternal outcomes, as we observed no significant differences in maternal bleeding outcomes.

Beyond the well‐studied benefit of magnesium for neuroprotection and maternal seizure prophylaxis, there is limited data on the neonatal effects of magnesium solely for seizure prophylaxis. The majority of the existing data is primarily focused on dose‐dependent relationships between magnesium sulfate and neonatal outcomes. In a study by Ghanavati et al., rates of neonatal hypotonia increased from 3% with maternal magnesium level ranging from 3.0 to 3.99 mEq/L to as high as 12% with maternal magnesium level 7.0 mEq/L or greater (*p* < 0.001). After adjusting for gestational age, nulliparity, and length of labor, intubation of the neonate in the delivery room was significantly associated with maternal serum magnesium level greater than 7.0 mEq/L (OR, 4.6; 95% CI, 1.4–15.4). While adverse outcomes increased with rising maternal serum magnesium level, the majority of neonatal effects occurred primarily with levels within the desired therapeutic range [13]. Similar results were also demonstrated by Chronnock et al., in which prolonged treatment with magnesium sulfate for >24 h in term neonates was associated with increased odds of NICU admission due to neonatal lethargy (aOR, 4.78; 95% CI, 1.5–15.21). The rate of NICU admission increased in a linear fashion with duration of maternal magnesium administration [14]. While both of these studies demonstrate neonatal adverse effects with prolonged magnesium exposure, they are limited by the absence of a control group that was not exposed to magnesium. We add to this literature by comparing neonatal outcomes in infants exposed to magnesium versus no magnesium. While we were unable to describe the exact indication for the NICU admission (i.e., hypotonia, lethargy, and respiratory distress), we demonstrated an increased rate of admission to the NICU in infants exposed to intrapartum magnesium for preeclampsia seizure prophylaxis. Additionally, there are limited data describing the rates of acidemia with magnesium sulfate administration, and thus, we add to the literature with the findings of decreased acidemia with magnesium administration and even a potential of decreased adverse neonatal outcomes when excluding NICU admission.

The results of our study are similarly supported by Greenberg et al. In this retrospective cohort study, NICU admission rates were compared between term neonates exposed to magnesium sulfate for preeclampsia prophylaxis and neonates born to individuals with preeclampsia who were not exposed to magnesium. Preeclampsia with severe features—a disorder with more severe neonatal effects—was not an exclusion criterion in this study and thus, groups were different in that more individuals in the magnesium‐exposed group had preeclampsia with severe features. NICU admission rate was 14.7% in magnesium‐exposed groups, compared to 5.1% in the groups not exposed to magnesium, corresponding to a number needed to harm of 11 for neonatal magnesium exposure per NICU admission [15]. It is well studied that the benefits of magnesium sulfate outweigh the risks in patients with severe features of preeclampsia. While in Greenberg et al., we are unable to tease out the effect of magnesium solely in patients without severe preeclamptic features, we add to the literature by describing similar findings of increased NICU admission rates in our population of patients with preeclampsia without severe features.

The focus of this study was primarily on neonatal outcomes, however, effects of magnesium on maternal bleeding were studied as a secondary outcome. We found no significant difference in hemorrhage (EBL > 1 L), transfusion requirement, or change in pre‐ and postdelivery hemoglobin. Our findings are consistent with findings in the MagPie trial and Dhillon et al., both of which fail to show a significant change in blood loss or postpartum hemorrhage with magnesium administration [5, 10].

At present, a universal practice for administration of magnesium for preeclampsia without severe features does not exist and ACOG defers the decision to the provider or institution, creating a wide range of practices. This raises the question of how best to standardize treatment guidelines for preeclampsia without severe features while considering both maternal and neonatal risks and benefits of intrapartum magnesium sulfate. Further prospective studies are warranted to clarify the potential risks and benefits of intrapartum magnesium for seizure prophylaxis administration to the neonate. The discordant findings of higher odds of NICU admission but a lower incidence of fetal acidemia and neonatal morbidity (excluding NICU) with magnesium administration warrant further investigation. As there are many factors that drive NICU admission, it is possible that NICU admission itself may not be a serious neonatal comorbidity as compared to neonatal acidemia (reduced in the magnesium group). While these findings could also additionally be contributed to type 2 error, further prospective studies are warranted to evaluate indication for NICU admission as well as long‐term neonatal outcomes as acidemia is a worse prognostic factor than NICU stay alone, without acidemia.

There are several strengths of our study including a large sample size with individual chart review as opposed to use of ICD codes—limiting preeclampsia misclassification bias (i.e., ensuring no preeclampsia with severe features was included)—and performance at a single institution with standardized care and practices, limiting variations of management of preeclampsia over the study period. We also acknowledge several limitations. Limitations of our study include the retrospective nature of or study and associated bias. As this study was retrospective, there may be additional differences between cohorts that we were unable to capture or adjust for, leading to potential residual, unmeasured confounding. Alternative adjustment methods, such as propensity score matching, could also be used. However, these approaches still cannot account for unmeasured confounding and are unlikely to change our findings, given the large sample size and adequate number of events in our study. We also acknowledge that for common outcomes, the use of ORs may overestimate the true association. However, with the rarity of other outcomes and the retrospective nature of the study, ORs were used in this analysis. Furthermore, while our study population is quite large, the study is not powered to detect rare outcomes such as neonatal death. For twin gestations, outcomes were analyzed per pregnancy, which we acknowledge may underestimate the outcome frequency. As a second limitation, we were also unable to assess the relationship between magnesium for seizure prophylaxis in neonates born <32 weeks because they may also receive magnesium for an alternative reason (neuroprotection). We also acknowledge missingness of data—as umbilical artery pH and base deficit had higher levels of missingness—which may introduce bias. As there are higher missing rates in the magnesium group, there is potential that they were missing due to inability to obtain a cord gas in the setting of an ill neonate. If represented, this could skew the findings toward greater neonatal morbidity than reported (i.e., toward the null hypothesis). Finally, we were unable to ascertain the indications for delivery or NICU admission, nor assess institution‐specific clinical practice patterns that may influence NICU admissions. Characteristics of magnesium use (duration of infusion, total dose, and maternal magnesium levels) were also not assessed in this study. We acknowledge that these factors, in addition to clinician judgment and practice style, may impact rates of NICU admission and other components of the neonatal composite. Further, this study took place at a large tertiary care referral center that includes a medically, socioeconomically, and geographically diverse population that is generally a high‐risk patient population that may not be generalizable to other populations. In sum, given the retrospective design, our findings demonstrate association rather than causation.

## 5 CONCLUSION

In conclusion, at our tertiary care center, magnesium sulfate administration in women with preeclampsia without severe features was associated with higher odds of adverse neonatal outcomes driven by NICU admission. However, when excluding NICU admission, magnesium was associated with a lower odds of adverse neonatal outcome as well as a lower incidence of fetal acidemia. Trials—with carefully evaluated short‐ and long‐term neonatal outcomes—should be conducted to confirm these results and create standardized treatment guidelines.

## CONFLICT OF INTEREST STATEMENT

The authors declare no conflicts of interest.

## FUNDING INFORMATION

The authors received no specific funding for this work.

## Data Availability

The data that support the findings of this study are available from the corresponding author upon reasonable request.
